# Pseudidiomarina xizangensis sp. nov. and Terrihabitans aquatilis sp. nov., isolated from LungmuCo lake in Xizang, with the reclassification of Flaviflagellibacter deserti as Terrihabitans deserti comb. nov.

**DOI:** 10.1099/ijsem.0.007073

**Published:** 2026-02-11

**Authors:** Zi-Xuan Liu, Rui Wang, You-Jun Liao, Dorji Phurbu, Ai-Hua Li

**Affiliations:** 1 China General Microbiological Culture Collection Center, Institute of Microbiology, Chinese Academy of Sciences, Beijing 100101, PR China; 2 School of Biotechnology and Food Science, Tianjin University of Commerce, Tianjin 300131, PR China; 3 Xizang Key Laboratory of Plateau Fungi, Institute of Plateau Biology of Xizang Autonomous Region, Lhasa 850001, PR China

**Keywords:** *Hyphomicrobiales*, *Idiomarinacea*, LungmuCo lake, *Pseudidiomarina*, *Terrihabitans*, Xizang

## Abstract

Two bacterial strains, designated E22-M8^T^ and B22-R8^T^, were isolated from water sample collected from LungmuCo lake in Xizang of China. The 16S rRNA gene analysis revealed that strain E22-M8^T^ was most closely related to members of the genus *Pseudidiomarina*, exhibiting the highest sequence similarity of 97.38 and 97.39% to *Pseudidiomarina halophila* BH195^T^ and *Pseudidiomarina terrestris* 1APP75-27a^T^, respectively. While strain B22-R8^T^ showed the highest similarity to species of the genera *Terrihabitans* and *Flaviflagellibacter*: 97.69, 96.80 and 96.66% to ‘*Terrihabitans rhizophilus*’ PJ23^T^, *Terrihabitans soli* IZ6^T^ and *Flaviflagellibacter deserti* SYSU D60017^T^. For strain E22-M8^T^, the digital DNA–DNA hybridization (dDDH) values with its closest relatives *P. halophila* BH195^T^ and *P. terrestris* 1APP75-27a^T^ were 25.7 and 20.1%, respectively; the average nucleotide identity (ANI) values were 83.5 and 78%; and the average amino acid identity (AAI) values were 90.4 and 86.4%, all below the recognized species delineation thresholds. For strain B22-R8^T^, the corresponding dDDH, ANI and AAI values with its closest relatives ‘*T. rhizophilus*’ PJ23^T^, *T. soli* IZ6^T^ and *F. deserti* SYSU D60017^T^ also fell below the respective species thresholds. Biochemically and physiologically, both strains exhibited distinct traits that further supported their novelty. Based on comprehensive polyphasic analyses, strain E22-M8^T^ (=CGMCC 1.19205^T^=KCTC 92346^T^) is proposed as a novel species named *Pseudidiomarina xizangensis* sp. nov., and strain B22-R8^T^ (=CGMCC 1.19187^T^=KCTC 92343^T^) as a novel species designated *Terrihabitans aquatilis* sp. nov.

## Introduction

The Qinghai-Xizang Plateau, with an average elevation of over 4,000 m, was known as the highest plateau on Earth [1]. Salt lakes in Xizang were mainly distributed in the northern part of the Xizang Plateau region, with nearly 500 discovered salt lakes of various sizes across the region. Compared to salt lakes in other areas, these lakes were characterized by high altitude, low temperature and limited nutrient availability. Additionally, some lakes were replenished by glacial melt and snowmelt, which contributes to the uniqueness of their environmental parameters. Sharpened by high altitude, low atmospheric pressure, harsh climatic conditions and poor accessibility, these regions harbour rich and distinctive biological resources [2].

The genus *Pseudidiomarina* belongs to the family *Idiomarinaceae* [3], the order *Alteromonadales* [4], the class *Gammaproteobacteria* and the phylum *Pseudomonadota*. It was first described by Jean *et al.* in 2006 [5], with the type species *Pseudidiomarina taiwanensis* PIT1^T^ isolated from shallow coastal waters. Since then, several species of this genus have been described, isolated from diverse global environments, including rhizosphere soil, surface sediment and seawater. To date, there are a total of 21 subtaxa within the genus, 19 of which have been validly published. The species *Pseudidiomarina halophila* BH195^T^, isolated from a solar saltern sediment, was first proposed by Lee as *Idiomarina halophila* [6], before being transferred to the genus *Pseudidiomarina* by Liu in 2019 [7].

The genus *Terrihabitans* was first proposed by Nakai *et al.* in 2022, with *Terrihabitans soli* IZ6^T^ as type species [8]. Although ‘*Terrihabitans rhizophilus*’ PJ23^T^ was subsequently proposed by Bao *et al.*, it has not yet been validly published according to List of Prokaryotic names with Standing in Nomenclature (LPSN) [9]. The genus *Flaviflagellibacter*, represented solely by *Flaviflagellibacter deserti* SYSU D60017^T^ [10], was a close phylogenetic relative of *Terrihabitans*. All these species were predominantly isolated from soil and plant roots and were classified within the order *Hyphomicrobiales*. Notably, the earliest described species of the genus *Terrihabitans* was initially assigned to the order *Rhizobiales* in earlier studies, but has been reclassified under *Hyphomicrobiales* following taxonomic revisions. Furthermore, the family-level classification of both *Terrihabitans* and *Flaviflagellibacter* has recently been updated to *Methylopilaceae* [11].

## Methods

### Isolation and preservation

Water samples were collected from LungmuCo lake (34° 32′ 31.34″ N 80° 25′ 14.16″ E), a high-altitude lake in Ngari Prefecture. The samples were immediately transported to the laboratory under a cold and dark environment. Water samples were subjected to gradient separated cultivation, and then many single colonies were obtained, purified and identified from marine agar 2216 medium and Reasoner’s 2A (R2A) medium after incubation at 25 °C for 7 days. Strain E22-M8^T^ was acquired from marine agar 2216 medium, while strain B22-R8^T^ was isolated from R2A medium. The isolated strains were subcultured on the fresh media, respectively, then preserved by lyophilization and stored in liquid nitrogen.

All reference type strains were obtained from China General Microbiological Culture Collection Center (CGMCC), including *P. halophila* BH195^T^=CGMCC 1.13913^T^, *F. deserti* SYSU D60017^T^=CGMCC 1.16444^T^ and ‘*T. rhizophilus*’ PJ23^T^=CGMCC 1.61577^T^.

### Phenotypic characteristics

Cell morphology was observed using a light microscope and transmission electron microscope (JEM1400, Japan; HT7800/Regulus8100, Japan) after 48 h of cultivation on their corresponding medium at 25 °C. The growth conditions of the novel strains, including the optimal growth temperature, salinity and pH, were determined following the method described previously [12]. Anaerobic growth ability was tested on their optimal growth medium with 0.1% (w/v) NaNO_3_, NaNO_2_ and FeCl_3_, respectively. Gram-staining, DNase, catalase and oxidase activity tests were conducted following standard protocols [13]. Physiological tests and carbon source utilization were carried out using the GEN III MicroPlate system (Biolog) and API ZYM, 20E, 20NE and 50CH (bioMérieux) test strips following the manufacturer’s instructions. Hydrolysis of substrates (5.0% casein, 1.0% cellulose, 1.0% Tween 20, 1.0% Tween 80, 1.0% starch, w/v) was evaluated on corresponding culture plates at 25 °C for 7 days. Related type spices were selected after a comprehensive analysis and comparison of the results of the phylogenetic tree based on 16S rRNA and phylogenomic tree.

### Chemotaxonomic analysis

The two novel strains were separately cultured on their respective optimal culture media. The strain E22-M8^T^ was grown on marine agar 2216 medium, while the strain B22-R8^T^ was cultured on R2A medium. For fatty acid profile analysis, novel strains were cultured on optimal culture medium for 48 h. Cells were harvested, and fatty acids were saponified, methylated and extracted according to standard protocols [14]. The extracted cellular fatty acids were identified and quantified by the Sherlock Microbial Identification System (MIDI) using a GC (Agilent 6890 N) [15]. For polar lipids and respiratory quinones tests, novel isolates were cultured in broth of optimal culture medium. After the strains reached the logarithmic growth phase, the bacteria were collected by centrifugation and then freeze-dried. Polar lipids were extracted, separated and identified following previous methods [16], and respiratory quinones were extracted, separated and analysed by HPLC [17,18].

### Phylogenetic analysis

The genomic DNA of strains E22-M8^T^ and B22-R8^T^ was extracted using the Genomic DNA Rapid Isolation Kit for bacterial cells (BioDev-Tech; Beijing, China) following the instructions of the manufacturer. The 16S rRNA gene sequence was amplified by PCR with a pair of universal primers 27F (5′-AGAGTTTGATCCTGGCTCAG-3′) and 1492R (5′-TACGGCTACCTTGTTACGACTT-3′) [19,20]. Sequencing was performed by Sanger sequencing, and the results were identified by EzBio-Cloud and GenBank databases, respectively [21]. Phylogenetic analysis based on 16S rRNA gene sequences was performed using MEGA 12 [22]. Multiple alignments were carried out with clustal W [23], and evolutionary distance matrices were calculated using the Kimura two-parameter model [24]. Phylogenetic trees were constructed by the neighbour-joining [25], maximum-likelihood [26] and maximum-parsimony methods [27], and the topology of the phylogenetic tree was evaluated based on 1,000 replications.

### Genomic sequencing and annotation

The genomic DNA of novel isolates was sequenced using the Illumina HiSeq X platform at the Majorbio Bio-Pharm Technology Co., Ltd. (Beijing, China). The raw data were processed to obtain clean data, which were then uploaded to the BV-BRC 3.47.11 (https://www.bv-brc.org/) for assembly and annotation [28,29]. Phylogenomic analysis of the novel strain together with closely related type strains was carried out using the Bacterial Genome Tree tool on the BV-BRC 3.47.11 platform. The phylogenetic tree was constructed based on single-copy genes, and the number of genes was set as 1,000. Average nucleotide identity (ANI) values were calculated using the ANI calculator tool (https://www.ezbiocloud.net/tools/ani) [30], and digital DNA–DNA hybridization (dDDH) values were calculated via the genome-to-genome distance calculator with formula 2 (https://ggdc.dsmz.de/ggdc.php) [31]. The average amino acid identity (AAI) values were obtained by estimating genomic datasets of proteins using Compare M. Draft genomes of novel isolates were annotated using the Genome Annotation tool NCBI Prokaryotic Annotation Pipeline. Functional annotation was performed by RAST (Rapid Annotation using Subsystems Technology) [32] and visualized via SEED viewer [33]. Biosynthetic gene clusters (BGCs) were predicted via antiSMASH (https://antismash.secondarymetabolites.org) to assess secondary metabolite properties [34].

## Results and discussion

The Qinghai-Tibet Plateau region features unique environmental conditions and rich biodiversity. Exploring the taxonomic composition, ecological adaptation mechanisms and biotechnological potential of micro-organisms in these extreme environments is of utmost urgency and thus fills gaps in our understanding of microbial biogeography and evolution in high-altitude aquatic habitats. During a systematic microbial diversity survey focused on high-altitude salt lakes in the Xizang Autonomous Region, China, water samples were collected from LungmuCo lake, a high-altitude lake in Ngari Prefecture. Numerous bacterial strains were isolated from various culture media, including marine agar 2216, R2A and R2A-modified media, to maximize the recovery of diverse taxa. Among these isolates, several strains have been subjected to taxonomic characterization and formally described, with six strains belonging to the genus *Algoriphagus* [35], the strain C22-A2^T^ assigned to *Virgibacillus tibetensis* [36] and three novel isolates assigned to the genus *Novilysobacter* [37]. The strains E22-M8^T^ and B22-R8^T^ investigated represent two additional novel taxa identified from this sampling effort, further expanding our knowledge of the unique microbial resources in Xizang’s high-altitude salt lakes.

### 16S rRNA gene sequence analysis

**Fig. 1.**
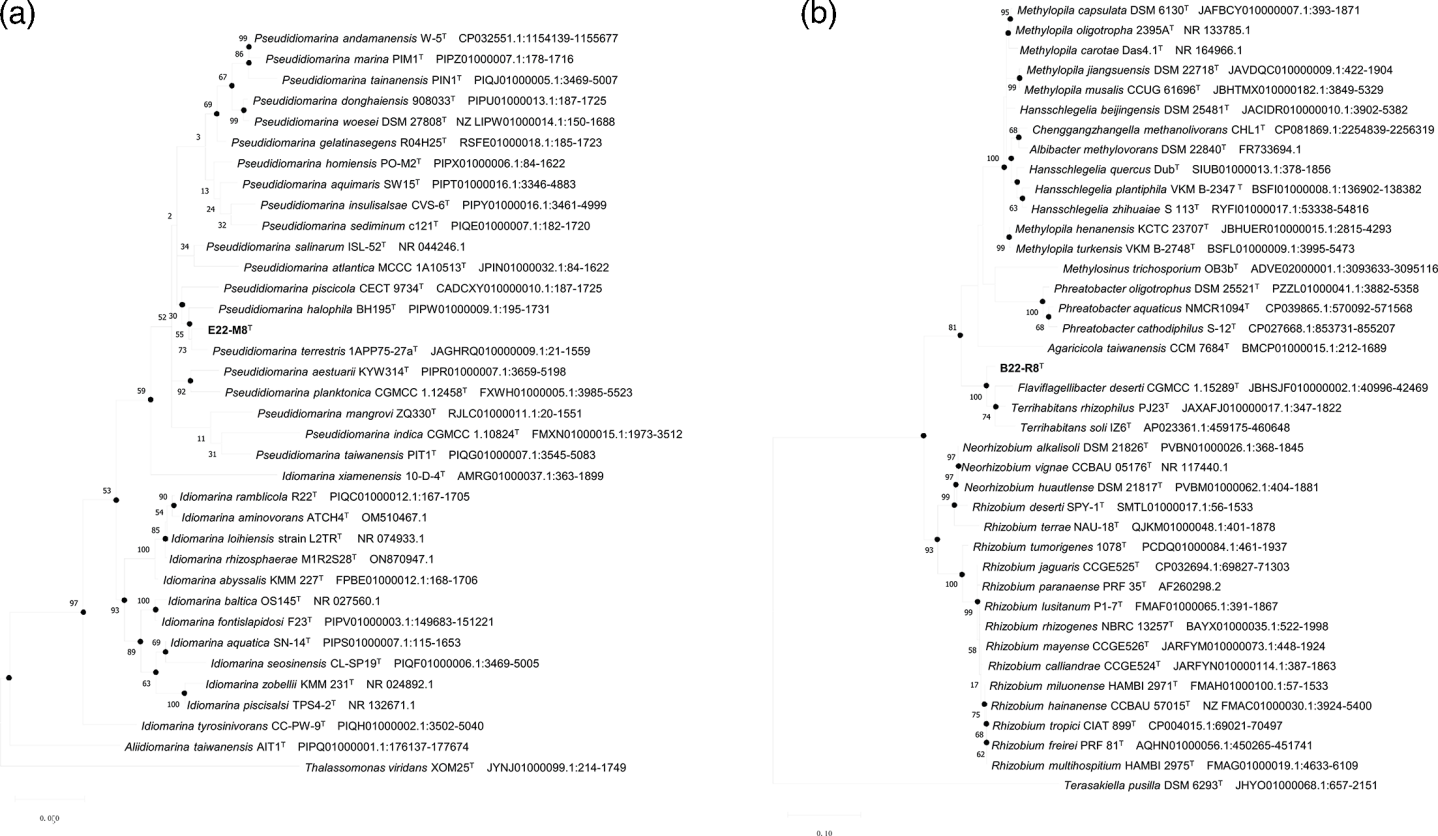
Maximum-likelihood phylogenetic tree based on 16S rRNA gene sequences of novel isolates and related taxa. Bootstrap values are expressed as percentages of 1,000 replications and shown at branching nodes (>50%). Genbank accession numbers are shown in parentheses. The filled circles indicate nodes recovered using the neighbour-joining method and maximum-parsimony methods. Bar, 0.05 substitution per nucleotide position. (a) Phylogenetic tree of 16S rRNA gene sequences using the K2+G+I model for strain E22-M8^T^. (b) Phylogenetic tree of 16S rRNA gene sequences using the K2+G+I model for strain B22-R8^T^.

Based on 16S rRNA gene sequence comparison, strain E22-M8^T^ exhibited the highest similarity to *Pseudidiomarina terrestris* 1APP75-27a^T^ (97.39%) and *P. halophila* BH195^T^ (97.38%). Given its relatedness to members of the genus *Pseudidiomarina*, the phylogenetic position of strain E22-M8^T^ was further evaluated. In the maximum-likelihood phylogenetic tree reconstructed from 16S rRNA gene sequences, strain E22-M8^T^ clustered together with *P. terrestris* 1APP75-27a^T^ and then formed a stable branch with *P. halophila* BH195^T^ (Fig. 1), supporting its identification as a novel species within the genus.

The 16S rRNA gene sequence of strain B22-R8^T^ exhibited the highest similarity (97.69%) to ‘*T. rhizophilus*’ PJ23^T^, followed by *T. soli* IZ6^T^ and *F. deserti* SYSU D60017^T^ (96.80 and 96.66% similarity, respectively). Phylogenetic analysis based on 16S rRNA gene further clarified the taxonomic position of strain B22-R8^T^, revealing that it formed a robust clade with the branch comprising ‘*T. rhizophilus*’ PJ23^T^, *F. deserti* SYSU D60017^T^ and *T. soli* IZ6^T^. These results suggested that strain B22-R8^T^ could represent a new species within the genus *Terrihabitans* or *Flaviflagellibacter* (Fig. 1). However, further studies were required to definitively determine its taxonomic position.

To verify the accuracy of the 16S rRNA gene sequence, the PCR-amplified fragments (GenBank accession no. PV668770 for E22-M8^T^; PV668771 for B22-R8^T^) were aligned with the 16S rRNA gene sequence derived from the corresponding whole-genome assemblies (JBOBQA000000000 for E22-M8^T^; JBOCUO000000000 for B22-R8^T^) based on the annotations from National Center for Biotechnology Information (NCBI). Alignment results of strain E22-M8^T^ showed 99.7% sequence identity (1,413/1,417) between the PCR-amplified 16S rRNA gene and the genome-derived counterpart. For strain B22-R8^T^, the sequence identity of PCR-amplified result and the corresponding genome-derived sequence was 99.9% (1,340/1,341). Collectively, these results confirmed the reliability of the PCR products and the integrity of the genome-derived 16S rRNA gene sequences.

### Whole-genome sequence analysis

**Fig. 2.**
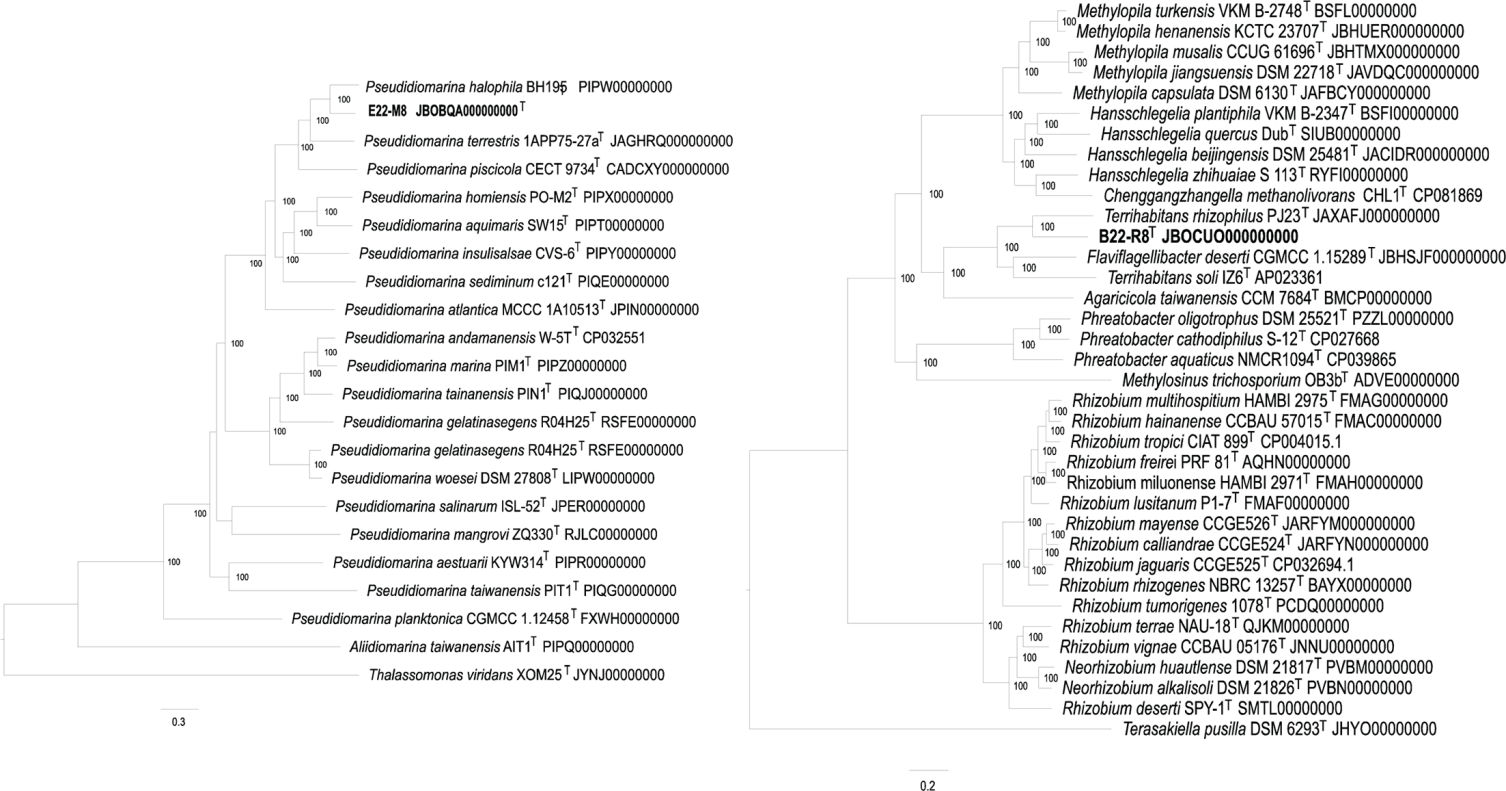
The phylogenomic tree is based on newly identified strains and related type species. (a) Phylogenomic tree of the strain E22-M8^T^. Bar, 0.3 nucleotide substitution rate (Knuc) units. (b) Phylogenomic tree of the strain B22-R8^T^. Bar, 0.2 nucleotide substitution rate (Knuc) units.

The genome of strain E22-M8^T^ comprised nine contigs, with a size of 2,460,956 bp and an N50 value of 1,553,895. The DNA G+C content was 49.3 mol%, as determined through genomic bioinformatics analysis. Genome completeness evaluated via CheckM was 99.7%. Phylogenomic analysis based on 695 single-copy genes revealed that strain E22-M8^T^ formed a distinct clade with *P. halophila* BH195^T^, which subsequently clustered with *P. terrestris* 1APP75-27a^T^ (Fig. 2).

The dDDH values between strain E22-M8^T^ and the reference type species *P. halophila* BH195^T^ and *P. terrestris* 1APP75-27a^T^ were 25.7 and 20.1%, respectively, both well below the 70% species delineation threshold. Similarly, ANI and AAI values with *P. halophila* BH195^T^ (83.5%, 90.4%) and *P. terrestris* 1APP75-27a^T^ (78%, 86.4%) also fell below the species delineation standard of 95–96% (Table 1). Based on the above result, it confirmed that *P. halophila* BH195^T^ was the closest relative of strain E22-M8^T^ and strongly supported the classification of E22-M8^T^ as a novel species of the genus *Pseudidiomarina*.

**Table 1.** ANI values for pairwise comparisons between the novel isolates E22-M8^T^, BH195^T^ and their phylogenetic related species Strains: 1, *P. halophila* BH195^T^; 2, *P. terrestris* 1APP75-27a^T^; 3, *F. deserti* SYSU D60017^T^; 4, ‘*T. rhizophilus*’ PJ23^T^; 5, *T. soli* IZ6^T^.

	1	2	3	4	5
**E22-M8^T^ (ANI/dDDH**)	83.5/25.7	78/20.1	–	–	–
**B22-R8^T^ (ANI/dDDH**)	–	–	74.5/19.6	77.1/20.1	73.2/19.0

The genome of strain B22-R8^T^ was composed of six contigs, with a size of 3,101,132 bp and an N50 value of 1,028,737. The DNA G+C content was 64.1 mol%. CheckM assessment indicated a genome completeness of 89.4%. A phylogenomic tree reconstructed from 230 highly conserved single-copy genes showed that strain B22-R8^T^ formed a distinct clade with ‘*T. rhizophilus*’ PJ23^T^, which subsequently clustered with a branch comprising *T. soli* IZ6^T^ and *F. deserti* SYSU D60017^T^ (Fig. 2). This topology was largely consistent with the 16S rRNA phylogenetic tree, confirming the stable clustering of strain B22-R8^T^ with these three type strains. Notably, strain B22-R8^T^ exhibited the closest affinity to ‘*T. rhizophilus*’ PJ23^T^, while *F. deserti* SYSU D60017^T^ was more closely related to *T. soli* IZ6^T^.

The dDDH, ANI and AAI values between strain B22-R8^T^ and its closest relatives species *F. deserti* SYSU D60017^T^, ‘*T. rhizophilus*’ PJ23^T^ and *T. soli* IZ6^T^ were 19.6%, 74.5%, 69.4%; 20.1%, 77.1%, 76.1%; and 19%, 73.2%, 69.2%, respectively. All values fell below the 70% (dDDH) and 95–96% (ANI/AAI) thresholds for species assignment (Table 1). These results strongly supported that strain B22-R8^T^ represented a novel species of genus *Terrihabitans* (Tables 1 and S1, available in the online Supplementary Material).

### Genome annotation

Strain E22-M8^T^ was predicted to harbour 2,268 coding sequences (CDSs), 51 tRNAs and 2 rRNAs. Genes associated with antibiotic resistance and virulence factors were identified through multiple database comparisons (Table S2). Functional categorization using the SEED subsystem revealed that the majority of genes were involved in amino acids and derivatives, protein metabolism and the biosynthesis of cofactors, vitamins, prosthetic groups and pigments (Fig. S1). Secondary metabolite analysis performed with antiSMASH predicted the presence of seven BGCs, including those for two ribosomally synthesized and post-translationally modified peptide-like compounds, one betalactone, one terpene precursor, one arylpolyene and one ectoine.

The genome of strain B22-R8^T^ was annotated with 3,023 CDSs, 55 tRNA genes and 3 rRNA genes. Additional genomic characteristics, protein features and specialized genes are summarized in Table S2. Subsystem category distribution of strain B22-R8^T^ was annotated into amino acids and derivatives, protein metabolism, as well as cofactors, vitamins, prosthetic groups and pigment synthesis (Fig. S1). Antismash analysis identified three secondary metabolite BGCs in strain B22-R8^T^, comprising two terpenes clusters and one cluster associated with hydrogen-cyanide and redox-cofactor synthesis.

### Morphological, biochemical and physiological tests

The colony of strain E22-M8^T^ was circular, smooth, opaque and grey-white. Cells were rod-shaped, with multiple polar flagella observed (Fig. S2). For carbon source utilization, E22-M8^T^ could utilize propionic acid, though its overall metabolic profile was limited. In contrast, it showed the same ability to utilize glucuronamide, acetoacetic acid and l-fucose, similar to *P. halophila* BH195^T^, but unlike *P. terrestris* 1APP75-27a^T^ *[38].* Both strains E22-M8^T^ and *P. halophila* BH195^T^ were capable of reducing nitrate to nitrite (Table 2). Acid production occurred from d-glucose and aesculin, with weak acid production from methyl-*β*-d-xylopyranoside and d-mannose.

**Table 2.** Differential characteristics between the novel strains and their most related phylogenetic neighbours Strains: 1, *P. xizangensis* sp. nov. E22-M8^T^; 2, *T. aquatilis* sp. nov. B22-R8^T^; 3, *P. halophila* BH195^T^; 4, *F. deserti* SYSU D60017^T^; 5, ‘*T. rhizophilus*’ PJ23^T^. Symbols: +, positive; −, negative; w, weakly positive. All data are acquired from this experiment.

	1	2	3	4	5
**Isolated source**	Water of the salt lake	Salt lake	Solar saltern sediment	Desert soil	Rhizosphere soil
**Growth conditions (Optimum**)					
Temperature (°C)	20–25 (25)	10–30 (25)	4–55 (30)	4–37 (28)	4–29 (24)
pH	8.0–9.0 (8.0–8.5)	6.0–9.0 (7.0–8.0)	3.5–10.5 (7.5)	5.0–8.0 (7.0)	6.0–8.6 (8.0)
NaCl (w/v, %)	3–15 (7–9)	0–2.0 (0–1)	0.5–11 (2–3)	0–1.5 (<0.5)	0–4.0 (2.5)
**Utilization of carbon resource**					
d-Melibiose	w	−	−	−	−
d-Fucose	w	−	−	−	−
l-Fucose	w	w	w	w	−
Glucuronamide	+	w	+	+	w
Acetoacetic acid	+	−	w	−	w
Propionic acid	w	−	−	−	−
l-Arginine	−	−	−	−	−
l-Serine	−	−	−	w	−
l-Aspartic acid	−	+	−	−	−
l-Glutamic acid	−	+	w	w	−
d-Aspartic acid	−	+	−	−	−
Glycerol	−	+	−	w	−
d-Malic acid	−	+	w	−	−
**Nitrate reduction**	+	−	+	−	−
**Hydrolysis of substance**					
Aesculin	+	−	+	−	−
Gelatin	−	−	−	−	−
Urease	−	−	−	−	−
**Enzyme activity**					
Leucine arylamidase	+	+	+	+	+
Valine arylamidase	+	w	+	+	+
Cystine arylamidase	+	−	+	−	+

The primary cellular fatty acids (>10%) of strain E22-M8^T^ were the summed feature 9 (iso-C_17:1_* ω*9*c* and/or 10-methyl C_16:0_), iso-C_15:0_ and iso-C_11:0_ 3-OH (Table 3). Similar to other *Pseudidiomarina* species, summed feature 9 was also detected at high levels in strain E22-M8^T^. However, notable differences were observed in the relative proportions of certain components, such as iso-C_17:0_ and iso-C_11:0_ 3-OH, compared to related strains. The predominant respiratory quinone was ubiquinone-8 (Q-8), a characteristic of the genus *Pseudidiomarina*. The polar lipid profile consisted of phosphatidylethanolamine (PE), diphosphatidylglycerol (DPG) and phosphatidylglycerol (PG) as major components, along with one aminolipid (AL), two phospholipids (PL) and three unidentified lipids (L). This profile distinguishes strain E22-M8^T^ from other species within the genus (Fig. S3). Further details regarding its physiological and biochemical characteristics are provided in Table 2 and the species description.

**Table 3.** Fatty acid composition of strain novel isolates and related species Strains: 1, *P. xizangensis* sp. nov. E22-M8^T^; 2, *T. aquatilis* sp. nov. B22-R8^T^; 3, *P. halophila* BH195^T^; 4, *F. deserti* SYSU D60017^T^; 5, ‘*T. rhizophilus*’ PJ23^T^. Fatty acids that represented under 1% for all the strains are omitted. Symbol: –, not detected or make up <1% of the total fatty acids. All data were acquired from this experiment.

	1	2	3	4	5
C_12:0_	–	0.3	–	1.4	0.1
iso-C_11:0_	3.5	–	2.7	–	–
iso-C_11:0_ 3-OH	12.8	–	10.7	–	–
iso-C_13:0_	1.7	–	2.0	–	–
iso-C_13:0_ 3-OH	5	–	4.6	–	–
iso-C_15:1_ F	3.8	–	5.1	–	–
iso-C_13:0_ 3-OH	18.6	–	26.5	–	–
C_16:0_	3.5	6.7	5.0	3.7	1.5
iso-C_17:0_	9.8	–	8.3	–	–
C_17:1_* ω*8*c*	0.4	1.1	0.6	0.7	0.5
C_17:0_	0.2	4.2	0.4	1.2	0.3
C_18:0_	0.5	2.0	0.3	0.5	0.4
C_20:1_* ω*7*c*	–	–	–	0.4	3.9
Cyclo C_19:0_* ω*8*c*	0.1	–	–	2.9	–
C_18:0_ 3-OH	–	1.3	–	–	0.3
*Summed feature					
1	0.1	1.2	0.1	0.3	–
2	0.1	3.9	–	3.8	2.0
3	2.1	3.8	5.5	3.4	9.5
8	2.1	71.6	1.7	78.8	78.2
9	29.4	–	20.9	–	–

*Summed feature represents two or three fatty acids that cannot be separated by the Microbial Identification system. Summed feature 1 represents iso-C_15:1_ H and/or C_13:0_ 3-OH. Summed feature 2 represents C_12:0_ aldehyde and/or unknown constituent. Summed feature 3 represents C_16:1_* ω*7*c*/C_16:1_* ω*6*c*. Summed feature 8 represents C_18:1_* ω*7*c* and/or C_18:1_* ω*6*c*. Summed feature 9 represents iso-C_17:1_* ω*9*c* /10-methyl C_16:0_.

Colonies of strain B22-R8^T^ were circular, smooth and translucent-white. The cells were oval-shaped, with a single polar flagellum clearly visible (Fig. S2). Comparative analysis of carbon source utilization revealed both shared and unique features among the tested strains. Strain B22-R8^T^ exhibited greater carbon source utilization capacity, such as l-glutamic acid and glycerol. Additionally, strain B22-R8^T^ was capable of utilizing l-aspartic acid (Table 2), a trait not observed in the reference strains. Enzyme activity profiling showed that strain B22-R8^T^, along with all related type strains except *T. soli* IZ6^T^, was positive for leucine arylamidase and valine arylamidase. Acid production occurred from d-arabinose, l-arabinose, d-ribose, d-xylose, l-xylose, d-galactose, d-glucose, d-mannose, melibiose and d-fucose.

The predominant cellular fatty acids of strain B22-R8^T^ were summed feature (C_18:1_* ω*7*c* and/or C_18:1_* ω*6*c*), accounting for over 70% of the total (Table 3). This profile was consistent with related species from the genera *Terrihabitans* and *Flaviflagellibacter*. The major respiratory quinone was ubiquinone-10 (Q-10), which is consistent with the reference species from genera *Terrihabitans* and *Flaviflagellibacter*. The major polar lipids consisted of DPG, phosphatidylcholine (PC), PE and PG (Fig. S3). Additionally, strain B22-R8^T^ contained three unidentified lipids (L1–3), which distinguished it from other type species. Further details regarding physiological and biochemical characteristics are provided in Table 2 and the species description.

## Conclusion

Strains E22-M8^T^ and B22-R8^T^, obtained from LungmuCo salt lake, could not be classified based on 16S rRNA gene sequences alone. To determine their taxonomic status, a comprehensive polyphasic study was conducted, including phylogenetic analyses based on 16S rRNA gene and whole-genome sequences, as well as detailed physiological and biochemical characterizations. Based on the results, strain E22-M8^T^ represents a novel species of the genus *Pseudidiomarina*, for which the name *Pseudidiomarina xizangensis* sp. nov. is proposed. Strain B22-R8^T^ is identified as a novel species of the genus *Terrihabitans*, for which the name *Terrihabitans aquatilis* sp. nov. is proposed.

Based on comprehensive polyphasic taxonomic analyses integrating phylogenomic, genomic similarity and evolutionary evidence, the type species of strain *F. deserti* SYSU D60017^T^, preliminarily associated with the genus *Flaviflagellibacter*, should be formally classified into the genus *Terrihabitans*. Phylogenomically, the strain formed a stable and well-supported monophyletic clade with recognized type strains of the genus *Terrihabitans*, including ‘*T. rhizophilus*’ PJ23^T^ and *T. soli* IZ6^T^, showing a much closer evolutionary relationship with each other. Genomically, comprehensive comparisons of the value of ANI, AAI and dDDH against established genus- and species-level taxonomic thresholds unequivocally demonstrate that *F. deserti* SYSU D60017^T^ is appropriately classified into the genus *Terrihabitans*.

## Description of *Pseudidiomarina xizangensis*

*Pseudidiomarina xizangensis* (xi.zang.en’sis. N.L. fem. adj. *xizangensis*, pertaining to Xizang, Tibet, referring to the geographical origin of the type strain).

Cells are Gram-stain-negative, strictly aerobic, motile with polar flagella and rod-shaped, 0.4–0.5 µm in width and 1.7–1.8 µm in length. Colonies are circular, smooth, non-transparent and grey-white coloured after incubating for 48 h at 25 °C on Marine agar 2216 (MA agar). Growth occurs at 20–25 °C (optimum, 25 °C), pH 8.0–9.0 (optimum, 8.0–8.5) and with 3–15% NaCl concentration (w/v; optimum, 7–9%). Catalase and oxidase positive. Hydrolyse Tween 20, but not casein, cellulose, starch or Tween 80. DNase activity is not present. Glucuronamide and acetoacetic acid are utilized as sole carbon resources, and d-melibiose, d-mannose, d-fucose, l-fucose, l-rhamnose, l-alanine, l-histidine, d-glucuronic acid, l-malic acid, propionic acid and acetic acid are weakly utilized. Positive for alkaline phosphatase, esterase (C4), esterase lipase (C8), leucine arylamidase, valine arylamidase, cystine arylamidase, trypsin, *α*-chymotrypsin, acid phosphatase and naphthol-AS-BI-phosphohydrolase. Acid production occurs from d-glucose and aesculin and weakly from methyl-*β*-d-xylopyranoside and d-mannose. Nitrate could be reduced to nitrite. Hydrolysis of aesculin is present. While it is negative for indole production, glucose fermentation, gelatin hydrolysis, o‑Nitrophenyl‑β‑D‑galactopyranoside (ONPG) and p‑Nitrophenyl‑β‑D‑galactopyranoside (PNPG) hydrolysis. The activities of urease, lysine decarboxylase, ornithine decarboxylase, tryptophan deaminase, citrate utilization, Voges–Proskauer test, H_2_S and indole production are negative. The predominant cellular fatty acids are summed feature 9 (iso-C_17:1_* ω*9*c* and/or 10-methyl C_16:0_), iso-C_15:0_ and iso-C_11:0_ 3-OH. The predominant ubiquinone is Q-8. The predominant polar lipids are DPG and PG.

The type strain E22-M8^T^ (=CGMCC 1.19205^T^=KCTC 92346^T^) was isolated from a water sample collected from LungmuCo salt lake. The GenBank/EMBL/DDBJ accession number for 16S rRNA gene of strain E22-M8^T^ is PV668770, and the DDBJ/ENA/GenBank accession number of the Whole-Genome Shotgun project of E22-M8^T^ is JBOBQA000000000. The genomic DNA G+C content is 49.3 mol% according to the genomic sequencing data.

## Description of *Terrihabitans aquatilis*

*Terrihabitans aquatilis* (a.qua’ti.lis. L. masc. adj. *aquatilis*, living in water).

Cells are Gram-stain-negative, strictly aerobic, oval-shaped with single polar flagella. Cell dimensions were 0.6–0.75 µm in width and 0.9–1.0 µm in length. Colonies on R2A after 48 h growth at 25 °C are circular, smooth and translucent-white in colour. Growth occurs at 10–30 °C (optimum, 25 °C), pH 6.0–9.0 (optimum, 7.5) and with additional NaCl concentration of 0–2% (w/v; optimum, 0–1%; w/v). Catalase and oxidase positive. Hydrolyse Tween 20, but does not hydrolyse casein, cellulose, starch, Tween 80 or DNase. For carbon source utilization, *α*-d-glucose, l-rhamnose, glycerol, d-aspartic acid, l-aspartic acid, l-glutamic acid, d-gluconic acid, l-lactic acid, d-malic acid and formic acid are used as sole carbon sources; dextrin, d-maltose, d-trehalose, d-cellobiose, sucrose, d-turanose, stachyose, d-raffinose and *α*-d-lactose are weakly utilized. Positive for esterase (C4), esterase lipase (C8), leucine arylamidase and naphthol-AS-BI-phosphohydrolase, and weakly positive for alkaline phosphatase, valine arylamidase and acid phosphatase. Acids are produced from d-arabinose, l-arabinose, d-ribose, d-xylose, l-xylose, d-galactose, d-glucose, d-mannose, melibiose and d-fucose. Negative for nitrate reduction, aesculin hydrolysis, arginine decarboxylase, gelatin hydrolysis, glucose fermentation, tryptophanase activity, indole production, Voges–Proskauer test, ONPG and PNPG hydrolysis, citrate utilization, H_2_S production, urease activity, lysine decarboxylase and ornithine decarboxylase activity. The major cellular fatty acids are summed feature 8 (C_18:1_* ω*7*c*/C_18:1_* ω*6*c*). The main respiratory quinone was Q-10. The major polar lipids consisted of DPG, PE, PG and PC.

The type strain is B22-R8^T^ (=CGMCC 1.19187^T^=KCTC 92343^T^), isolated from water sample collected at LungmuCo salt lake. The GenBank/EMBL/DDBJ accession number for 16S rRNA gene of strain B22-R8^T^ is PV668771. The draft genome has been deposited under accession number JBOCUO000000000. Genomic DNA G+C content was 64.1 mol%.

## Description of *Terrihabitans deserti* comb. nov.

*Terrihabitans deserti* (de.ser’ti. L. gen. n. *deserti*, of or pertaining to a desert).

Basonym: *Flaviflagellibacter deserti* Dong *et al.* 2019.

The description is identical to that given for *F. deserti* by Dong *et al.* [10]. The type strain, SYSU D60017^T^ (=CGMCC 1.16444^T^=NBRC 112958^T^), was isolated from a desert sample in Xinjiang, China. The DNA G+C content of the type strain is 63.8 mol% (determined from the draft genome).

## Supplementary material

10.1099/ijsem.0.007073 Uncited Supplementary Material 1.

## Abbreviations

AAI: average amino acid identity
ANI: average nucleotide identity
BGCs: biosynthetic gene clusters
CDS: coding sequence
CGMCC: China General Microbiological Culture Collection Center
dDDH: digital DNA–DNA hybridization
DPG: diphosphatidylglycerol
PC: phosphatidylcholine
PE: phosphatidylethanolamine
PG: phosphatidylglycerol
R2A: Reasoner’s 2A

## References

[1] Wu T (2001). The Qinghai-Tibetan plateau: how high do Tibetans live?. High Alt Med Biol.

[2] Ge W, Lin H, Xinying T (2021). Surface radiation characteristics of the Ali area, Northern Tibetan Plateau. Environ Res Commun.

[3] Ivanova EP, Flavier S, Christen R (2004). Phylogenetic relationships among marine Alteromonas-like proteobacteria: emended description of the family *Alteromonadaceae* and proposal of *Pseudoalteromonadaceae* fam. nov., *Colwelliaceae* fam. nov., *Shewanellaceae* fam. nov., *Moritellaceae* fam. nov., *Ferrimonadaceae* fam. nov., *Idiomarinaceae* fam. nov. and *Psychromonadaceae* fam. nov. Int J Syst Evol Microbiol.

[4] Bowman JP, McMeekin TA, Brenner DJ, Krieg NR, Staley JT, Garrity GM (2005). Bergey’s Manual of Systematic Bacteriology, Second Edition.

[5] Jean WD, Shieh WY, Chiu HH (2006). *Pseudidiomarina taiwanensis* gen. nov., sp. nov., a marine bacterium isolated from shallow coastal water of An-Ping Harbour, Taiwan, and emended description of the family *Idiomarinaceae*. Int J Syst Evol Microbiol.

[6] Lee JC, Kim YS, Yun BS, Whang KS (2015). *Idiomarina halophila* sp. nov., isolated from a solar saltern sediment. Int J Syst Evol Microbiol.

[7] Liu Y, Lai Q, Shao Z (2018). Genome-based analysis reveals the taxonomy and diversity of the family *Idiomarinaceae*. Front Microbiol.

[8] Nakai R, Naganuma T, Tazato N, Kunihiro T, Morohoshi S (2021). Characterization of *Terrihabitans soli* gen. nov., sp. nov., a Novel 0.2 μm-filterable soil bacterium belonging to a widely distributed lineage of *Hyphomicrobiales* (Rhizobiales). Diversity.

[9] Bao R, Guo H, Liang Y, Tang K, Feng F (2024). *Terrihabitans rhizophilus* sp. nov., isolated from the rhizosphere soil of plant in temperate semi-arid steppe. Antonie Van Leeuwenhoek.

[10] Dong L, Han M-X, Wang D, Liu F, Asem MD (2019). *Flaviflagellibacter deserti* gen. nov., sp. nov., a novel member of the order *Rhizobiales* isolated from a desert soil. Antonie Van Leeuwenhoek.

[11] diCenzo GC, Yang Y, Young JPW, Kuzmanović N (2024). Refining the taxonomy of the order *Hyphomicrobiales* (*Rhizobiales*) based on whole genome comparisons of over 130 type strains. Int J Syst Evol Microbiol.

[12] Liu Z-X, Phurbu D, Liu H-C, Zhou Y-G, Li A-H (2020). *Craterilacuibacter sinensis* gen. nov. sp. nov., isolated from a crater lake in China. Int J Syst Evol Microbiol.

[13] Gerhardt P, Murray RGE, Wood WA, Krieg NR (1994). Methods for General and Molecular Bacteriology.

[14] Miller LT (1982). Single derivatization method for routine analysis of bacterial whole-cell fatty acid methyl esters, including hydroxy acids. J Clin Microbiol.

[15] Sasser M (1990). MIDI Technical Note.

[16] Minnikin DE, Bolton RC, Hartmann S, Besra GS, Jenkins PA (1993). An integrated procedure for the direct detection of characteristic lipids in tuberculosis patients. Ann Soc Belg Med Trop.

[17] Collins MD, Goodfellow M, Minnikin DE (1985). Chemical Methods in Bacterial Systematics.

[18] Wu C, Lu X, Qin M (1989). Microbiology [English Translation of Microbiology.

[19] Karlson U, Dwyer DF, Hooper SW, Moore ER, Timmis KN (1993). Two independently regulated cytochromes P-450 in a *Rhodococcus rhodochrous* strain that degrades 2-ethoxyphenol and 4-methoxybenzoate. J Bacteriol.

[20] Lane DJ, Goodfellow M, Chichester E (1991). Nucleic Acid Techniques in Bacterial Systematics Chichester.

[21] Yoon S-H, Ha S-M, Kwon S, Lim J, Kim Y (2017). Introducing EzBioCloud: a taxonomically united database of 16S rRNA gene sequences and whole-genome assemblies. Int J Syst Evol Microbiol.

[22] Kumar S, Stecher G, Suleski M, Sanderford M, Sharma S (2024). MEGA12: Molecular Evolutionary Genetic Analysis Version 12 for adaptive and green computing. Mol Biol Evol.

[23] Larkin MA, Blackshields G, Brown NP, Chenna R, McGettigan PA (2007). Clustal W and Clustal X version 2.0. Bioinformatics.

[24] Kimura M (1980). A simple method for estimating evolutionary rates of base substitutions through comparative studies of nucleotide sequences. J Mol Evol.

[25] Saitou N, Nei M (1987). The neighbor-joining method: a new method for reconstructing phylogenetic trees. Mol Biol Evol.

[26] Felsenstein J (1981). Evolutionary trees from DNA sequences: a maximum likelihood approach. J Mol Evol.

[27] Fitch WM (1971). Toward defining the course of evolution: minimum change for a specific tree topology. Syst Zool.

[28] Davis JJ, Wattam AR, Aziz RK, Brettin T, Butler R (2020). The PATRIC bioinformatics resource center: expanding data and analysis capabilities. Nucleic Acids Res.

[29] Wick RR, Judd LM, Gorrie CL, Holt KE (2017). Unicycler: resolving bacterial genome assemblies from short and long sequencing reads. PLoS Comput Biol.

[30] Yoon SH, Ha SM, Lim JM, Kwon SJ, Chun J (2017). A large-scale evaluation of algorithms to calculate average nucleotide identity. Antonie Van Leeuwenhoek.

[31] Meier-Kolthoff JP, Carbasse JS, Peinado-Olarte RL, Göker M (2022). TYGS and LPSN: a database tandem for fast and reliable genome-based classification and nomenclature of prokaryotes. *Nucleic Acids Res*.

[32] Aziz RK, Bartels D, Best AA, DeJongh M, Disz T (2008). The RAST server: rapid annotations using subsystems technology. BMC Genomics.

[33] Overbeek R, Olson R, Pusch GD, Olsen GJ, Davis JJ (2014). The SEED and the rapid annotation of microbial genomes using subsystems technology (RAST). Nucleic Acids Res.

[34] Blin K, Shaw S, Augustijn HE, Reitz ZL, Biermann F (2023). antiSMASH 7.0: new and improved predictions for detection, regulation, chemical structures and visualisation. Nucleic Acids Res.

[35] Zheng YY, Zhang X, Liu ZX, Wang R, Phurbu D (2025). *Algoriphagus aurantiacus* sp. nov. and *Algoriphagus persicinus* sp. nov., two novel species isolated from the shore soil of salt lake. Int J Syst Evol Microbiol.

[36] Li D, Phurbu D, Zhang X, Liu Z-X, Wang R (2024). *Virgibacillus tibetensis* sp. nov., isolated from salt lake on the Tibetan plateau of China. Int J Syst Evol Microbiol.

[37] Phurbu D, Zhang X, Wang R, Liu Z-X, Zheng Y-Y (2025). *Novilysobacter viscosus* sp. Nov. and *Novilysobacter longmucuonensis* sp. Nov., two Nov.l species capable of producing proteases and RiPPs. BMC Microbiol.

[38] Galisteo C, de la Haba RR, Ventosa A, Sánchez-Porro C (2024). The hypersaline soils of the odiel saltmarshes natural area as a source for uncovering a new taxon: *Pseudidiomarina terrestris* sp. nov. Microorganisms.

