# Supplementary material for: Pseudidiomarina xizangensis sp. nov. and Terrihabitans aquatilis sp. nov., isolated from LungmuCo lake in Xizang, with the reclassification of Flaviflagellibacter deserti as Terrihabitans deserti comb. nov
Source: Int J Syst Evol Microbiol. 2026 Feb 11;76(2):007073. doi: 10.1099/ijsem.0.007073 (PMC12893665; doi:10.1099/ijsem.0.007073)
Supplement: Uncited Supplementary Material 1. [file ijsem-76-07073-s001.pdf]

Pseudidiomarina xizangensis **sp. nov., and** ***Terrihabitans aquatilis*** **sp. nov. isolated from LungmuCo lake in Xizang, with the reclassification of** ***Flaviflagellibacter deserti*** **as** ***Terrihabitans deserti*** **comb. nov.**

Zi-Xuan Liu[1], Rui Wang[1,2], You-Jun Liao[1,2], Dorji Phurbu[3], Ai-Hua Li[1*]

1. China General Microbiological Culture Collection Center, Institute of Microbiology, Chinese Academy of Sciences, Beijing 100101, PR China

2. School of Biotechnology and Food Science, Tianjin University of Commerce, Tianjin, PR China

3. Xizang Key Laboratory of Plateau Fungi, Institute of Plateau Biology of Xizang Autonomous Region, Lhasa, 850001, PR China

Corresponding author:

Ai-Hua Li: Email: liah@im.ac.cn; Tel: 86-10-6480-6073

Running title: *Pseudidiomarina xizangensis*; *Terrihabitans aquatilis*

Subject category: New Taxa; Subsection: *Pseudomonadota; Terrihabitans*

Keywords: *Pseudidiomarina, Terrihabitans,* LungmuCo lake, *Idiomarinaceae*, *Hyphomicrobiales*

Abbreviations: ANI, average nucleotide identity; CDS, coding sequence; CGMCC, China General Microbiological Culture Collection Center; dDDH, digital DNA–DNA hybridization; DSMZ, Deutsche Sammlung von Mikroorganismen und Zellkulturen; GGDC, Genome-to-Genome Distance Calculator; R2A, Reasoner's 2A; RiPP-like, Other unspecified ribosomally synthesised and post-translationally modified peptide product (RiPP); TEM, transmission electron microscopy; DPG, diphosphatidylglycerol; PG, phosphatidylglycerol; PE, phosphatidylethanolamine; PC, phosphatidylcholine; AL, unidentified aminolipid; PL, unidentified phospholipid; L, unidentified lipids.

Author Notes:

The GenBank/EMBL/DDBJ accession numbers for 16S rRNA gene of strains E22-M8^T and B22-R8^T are PV668770 and PV668771. The Whole Genome Shotgun project of E22-M8^T and B22-R8^T had been deposited at DDBJ/ENA/GenBank under accession numbers JBOBQA000000000 and JBOCUO000000000.

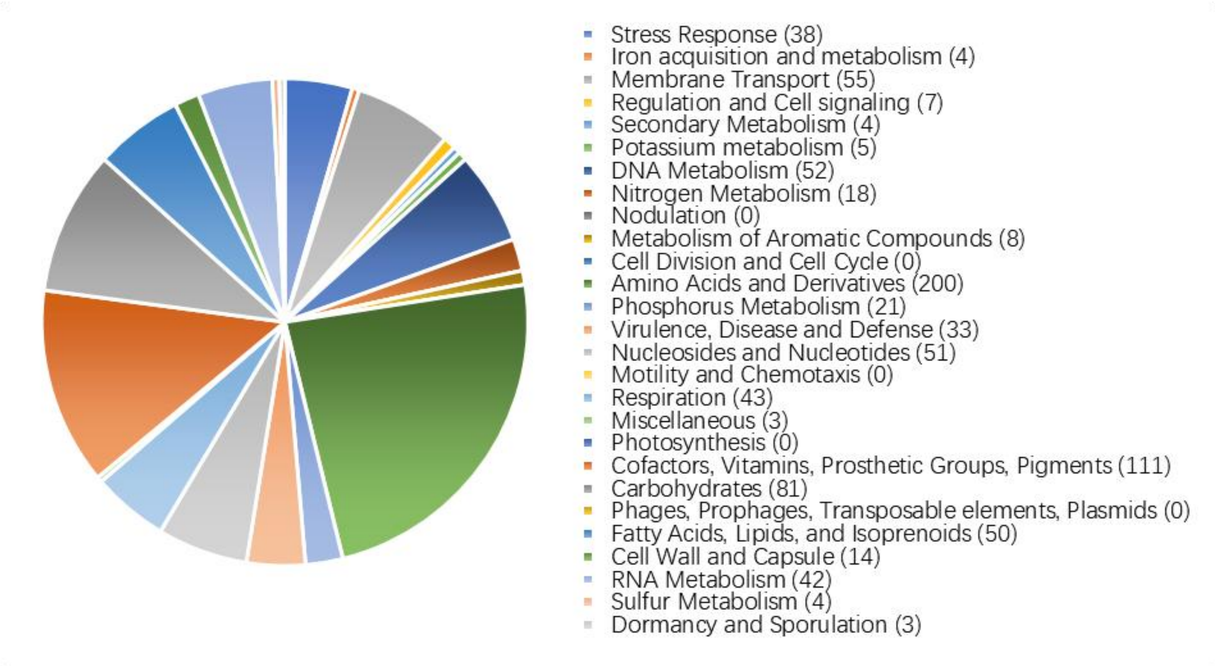

A

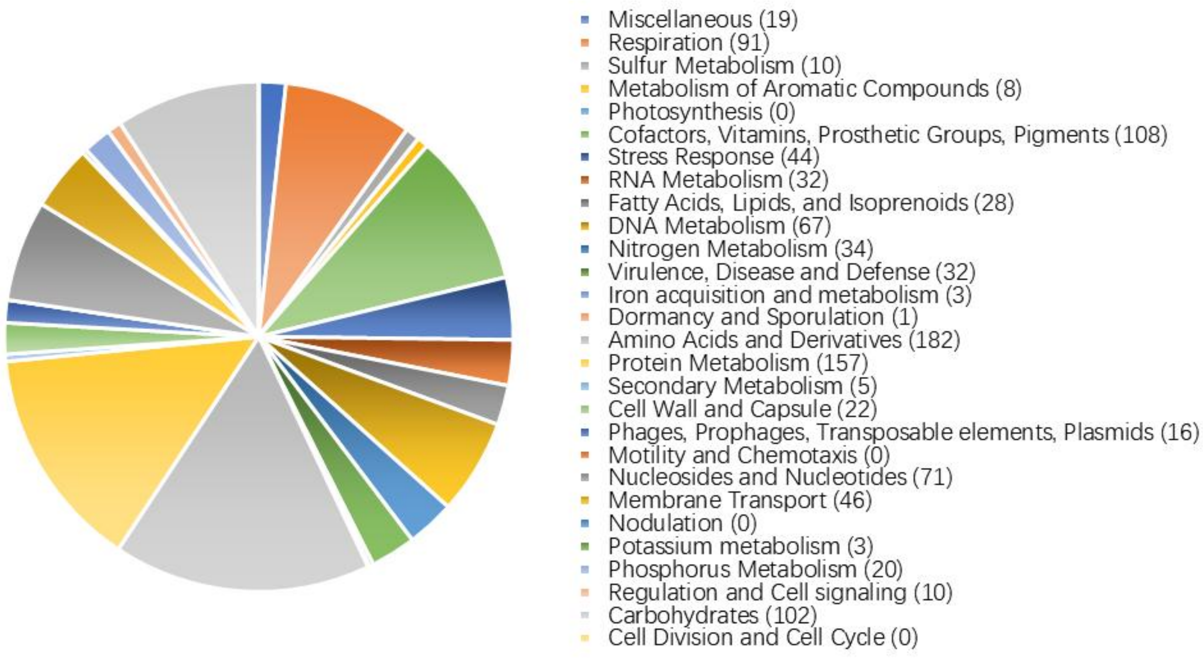

B

Figure S1: The subsystem category number of genes in novel strains detected by RAST annotation server. A: Subsystem category distribution of strain E22-M8^{T}. B: Subsystem category distribution of strain B22-R8^{T}.

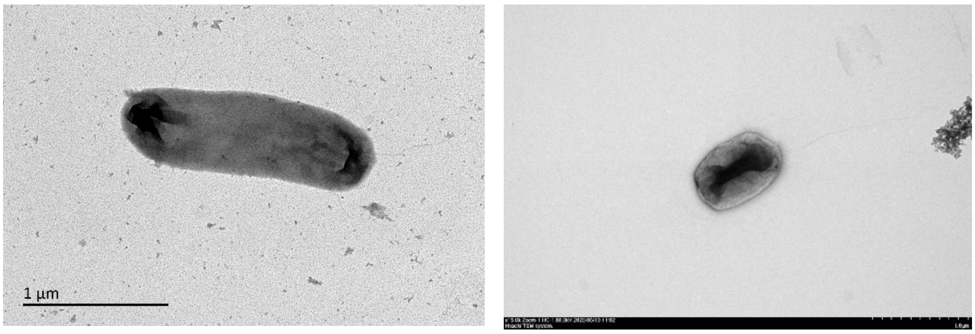

A B

Figure S2. Transmission electron microscope of strain E22-M8^T which were cultivated on MA agar at 25 ℃ for 48h was observed by JEL1400. Transmission electron microscope of strain B22-R8^T which were cultivated on R2A agar at 25 ℃ for 48h was observed by HT7800 TEM/Reg ulus8100.

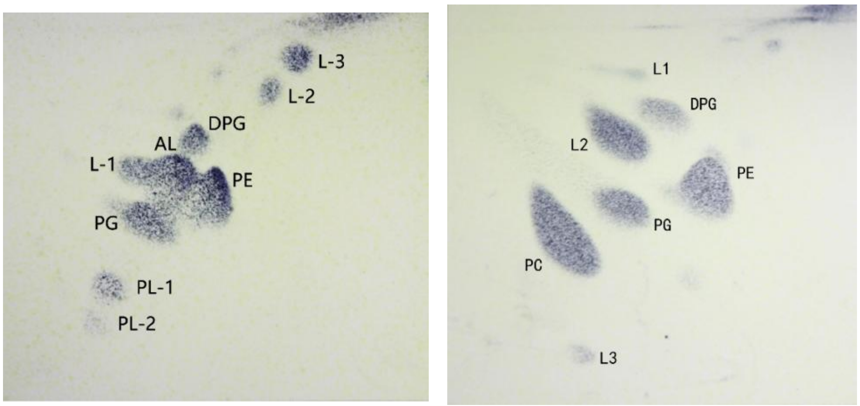

A B

Figure S3: The polar lipids of novel strains E22-M8^T (A) and B22-R8^T (B). Strain E22-M8^T and B22-R8^T were cultured on MA medium and R2A medium at 25 ℃ for 3 days respectively. Total polar lipids separated by two-dimensional TLC and detected with molybdophosphoric acid solution. Abbreviations: PG, phosphatidylglycerol；DPG, diphosphatidylglycerol； PE, phosphatidylethanolami； PL1-2, unknown phospholipids； AL, unknown aminolipid; L1-3, unknown lipids.

Table S1. A: AAI values for pairwise comparisons between the novel isolate and related the type species are shown below the diagona. AAI values equal to 100% are shaded gray.

**AAI**	P. tainanensis PIN1	P. terrestris 1APP75-27a	P. mangrovi ZQ330	**E22-M8**	P. marina PIM1	P. indica CGMCC 1.10824	P. insulisalsae CVS-6	P. planktonica CGMCC 1.12458	P. homiensis PO-M2	P. andamanensis W-5T	P. gelatinasegens R04H25	P. salinarum ISL-52	P. halophila BH195	P. woesei DSM 27808	P. piscicola CECT 9734	P. sediminum c121	P. aquimaris SW15	P. donghaiensis 908033	P. taiwanensis PIT1	P. atlantica MCCC 1A10513	P. aestuarii KYW314
P. tainanensis PIN1	100	73.3	72.1	**72.9**	91.6	78.5	72.8	68.7	73.6	91.6	84.7	73.4	73.2	83	72.9	72.6	73.8	83	71.3	73.3	73.2
P. terrestris 1APP75-27a	73.3	100	70.5	**86.3**	73.1	71.2	80	68.4	80.4	73.1	73.2	72.7	86.7	72.9	83.9	78	80.1	73	70.2	79	71.9
P. mangrovi ZQ330	72.1	70.5	100	**70.3**	72.2	71	70.4	67.9	70.8	72.1	72.1	72.5	70.6	72.2	70.1	70.6	70.9	72.2	70.4	70.7	71.7
**E22-M8**	**72.9**	**86.3**	**70.3**	100	**73.2**	**71**	**79.9**	**68.5**	**80.1**	**73**	**73**	**72.7**	**90.4**	**72.7**	**83.7**	**78.2**	**79.9**	**72.8**	**70.1**	**79.1**	**71.9**
P. marina PIM1	91.6	73.1	72.2	**73.2**	100	78.4	72.8	68.6	73.7	94	84.9	73.6	73	83.1	72.7	72.7	73.6	83.1	71.5	73.3	73.2
P. indica CGMCC 1.10824	78.5	71.2	71	**71**	78.4	100	71.1	67.9	71.4	78.5	77.2	71.7	71.3	76.8	70.7	70.9	71.5	76.7	70.5	71.3	71.2
P. insulisalsae CVS-6	72.8	80	70.4	**79.9**	72.8	71.1	100	68.4	82.6	72.7	72.6	72.7	79.8	72.5	78.7	79.3	82.9	72.5	70.6	78.7	71.6
P. planktonica CGMCC 1.12458	68.7	68.4	67.9	**68.5**	68.6	67.9	68.4	100	68.3	68.7	68.6	69.1	68.6	68.7	68.1	68.4	68.6	68.7	67.7	68.3	68.6
P. homiensis PO-M2	73.6	80.4	70.8	**80.1**	73.7	71.4	82.6	68.3	100	73.5	73.6	72.5	80.6	73.5	79.4	79.5	89.3	73.5	70.8	80.2	71.9
P. andamanensis W-5T	91.6	73.1	72.1	**73**	94	78.5	72.7	68.7	73.5	100	84.9	73.6	73.1	83.1	72.7	72.7	73.5	83.1	71.5	73.1	73.2
P. gelatinasegens R04H25	84.7	73.2	72.1	**73**	84.9	77.2	72.6	68.6	73.6	84.9	100	73.4	73.1	83	72.6	72.7	73.7	82.9	71.4	73.6	73
P. salinarum ISL-52	73.4	72.7	72.5	**72.7**	73.6	71.7	72.7	69.1	72.5	73.6	73.4	100	72.9	73.2	71.9	72.1	72.6	73.2	70.6	72.2	73
P. halophila BH195	73.2	86.7	70.6	**90.4**	73	71.3	79.8	68.6	80.6	73.1	73.1	72.9	100	72.8	83.5	77.9	80.1	72.8	70.2	78.8	72
P. woesei DSM 27808	83	72.9	72.2	**72.7**	83.1	76.8	72.5	68.7	73.5	83.1	83	73.2	72.8	100	72.5	72.7	73.3	95.2	71.3	73	73
P. piscicola CECT 9734	72.9	83.9	70.1	**83.7**	72.7	70.7	78.7	68.1	79.4	72.7	72.6	71.9	83.5	72.5	100	77.7	79.4	72.6	70.1	78.3	71.2
P. sediminum c121	72.6	78	70.6	**78.2**	72.7	70.9	79.3	68.4	79.5	72.7	72.7	72.1	77.9	72.7	77.7	100	79.2	72.5	70.6	77.7	71.4
P. aquimaris SW15	73.8	80.1	70.9	**79.9**	73.6	71.5	82.9	68.6	89.3	73.5	73.7	72.6	80.1	73.3	79.4	79.2	100	73.4	70.6	80.2	71.8
P. donghaiensis 908033	83	73	72.2	**72.8**	83.1	76.7	72.5	68.7	73.5	83.1	82.9	73.2	72.8	95.2	72.6	72.5	73.4	100	71.1	73	73
P. taiwanensis PIT1	71.3	70.2	70.4	**70.1**	71.5	70.5	70.6	67.7	70.8	71.5	71.4	70.6	70.2	71.3	70.1	70.6	70.6	71.1	100	70.5	72.1
P. atlantica MCCC 1A10513	73.3	79	70.7	**79.1**	73.3	71.3	78.7	68.3	80.2	73.1	73.6	72.2	78.8	73	78.3	77.7	80.2	73	70.5	100	72.1
P. aestuarii KYW314	73.2	71.9	71.7	**71.9**	73.2	71.2	71.6	68.6	71.9	73.2	73	73	72	73	71.2	71.4	71.8	73	72.1	72.1	100

B: The novel isolate B22-R8^T and all the type species of genus *Terrihabitans* and *Flaviflagellibacter*. SYSU D60017^T, *Flaviflagellibacter deserti* SYSU D60017^T; PJ23^T, *Terrihabitans rhizophilus* PJ23^T; IZ6^T, *Terrihabitans soli* IZ6^T.

AAI	B22-R8^T	SYSU D60017^T	PJ23^T	IZ6^T
B22-R8^T		69.41	76.06	69.25
SYSU D60017^T	69.41		69.43	70.76
PJ23^T	76.06	69.43		69.55
IZ6^T	69.25	70.76	69.55	

Table S2. General genome characteristics and annotation results of novel isolates E22-M8^T and B22-R8^T.

	**E22-M8^T**	**B22-R8^T**
**Genome accession number**	JBOBQA000000000	JBOCUO000000000
**Annotation information**		
Genes (total)	2325	3078
CDSs (total)	2268	3023
Genes (RNA)	57	55
rRNAs (5S, 16S, 23S)	0,1,1	1,1,1
tRNAs	51	48
ncRNAs	4	4
**Special Genes**		
Virulence Factor（Victors）	8	5
Virulence Factor（PATRIC_VF）	3	1
Virulence Factor（VFDB）	0	1
Transporter（TCDB）	4	4
Drug target（DrugBank）	14	2
Drug target (TTD)	3	0
Antibiotic resistance (PATRIC)	24	26
Antibiotic resistance (CARD)	2	0
